# Aging, Eye Movements, and Object-Location Memory

**DOI:** 10.1371/journal.pone.0033485

**Published:** 2012-03-12

**Authors:** Shui-I Shih, Katie L. Meadmore, Simon P. Liversedge

**Affiliations:** School of Psychology, University of Southampton, Southampton, United Kingdom; University of Leicester, United Kingdom

## Abstract

This study investigated whether “intentional” instructions could improve older adults' object memory and object-location memory about a scene by promoting object-oriented viewing. Eye movements of younger and older adults were recorded while they viewed a photograph depicting 12 household objects in a cubicle with or without the knowledge that memory about these objects and their locations would be tested (intentional vs. incidental encoding). After viewing, participants completed recognition and relocation tasks. Both instructions and age affected viewing behaviors and memory. Relative to incidental instructions, intentional instructions resulted in more accurate memory about object identity and object-location binding, but did not affect memory accuracy about overall positional configuration. More importantly, older adults exhibited more object-oriented viewing in the intentional than incidental condition, supporting the environmental support hypothesis.

## Introduction

Older adults often perform worse than younger adults in a range of episodic memory tasks and normal aging appears to affect episodic memory for associations between items more than for individual items [1]–[3]. According to Craik [1], such age-related deficits in memory can be “characterized as inefficiencies of processing, rather than as true losses or breakages of mechanisms or structure” (p. 350). He suggested that memory performance of older adults can benefit from proper environmental supports, of which an essential part is delivered by instructions that encourage (self-initiated) constructive operations (see also [4]). The instructions influence how one interacts with aspects of the environment. Thus, variations in memory performance would be better appreciated if one takes into account the dynamic and interactive nature of remembering [1]. The present research tested this environmental support hypothesis in the context of object-location memory and took eye movement measures to examine how instructions affect viewing behaviors which, in turn, affect performance in memory tasks.

Remembering the location of objects in our environment (i.e., object-location memory) is crucial for many daily tasks, for example, to find the medicine in a cupboard or to find a pair of glasses for reading. Object-location memory involves not only objects and spatial locations but also the association (or binding) between objects and locations [5], [6]. Research has shown poorer object-location memory in older than younger adults [6]–[10]. A prerequisite for forming object-location memory is to view the environment of interest. Although overt eye movements may dissociate from covert attention [11], people normally attend where they are looking [12]–[14] and neural networks for eye movements and covert attention appear to have substantial overlap [15]. Furthermore, eye movement behaviors have been related to memory for aspects of object identity and location [16]–[18] and short and longer term memory for scenes [16]–[20].

For example, Williams et al. [21] recorded eye movements of younger and older adults while they were counting the number of instances of a specified target (e.g., a yellow drill) among distractors (e.g., yellow telephone, red drill, and green door) displayed on a computer screen. Participants were then given a surprise recognition task (i.e., an incidental memory test) to assess their object identity memory for both targets and distractors that had appeared in the photographs. They found that the older adults made more and longer fixations and, thus, more fixations on the targets and distractors than the younger adults. Yet recognition accuracy was poorer in the older than younger adults. Critically, they found that recognition accuracy was positively associated with number of viewings and the total viewing time that the object had received, and the impact of additional viewing on object memory was similar for the younger and older age groups. However, had the older adults known about the test, it could be argued that they might have adopted a more suitable viewing strategy to improve related memory.

Indeed, memory performance is generally better under intentional than incidental learning conditions, especially for the older adults [1], [10]. For example, Uttl and Graf (1993, Experiment 2) assessed object-location memory of younger and older adults after they had interacted with objects in an office [10]. They manipulated instructions (intentional vs. incidental) so that participants were with or without the knowledge of a subsequent memory test. During the test, participants were asked to relocate objects back to their original locations in the office or to indicate their locations in an office map. As expected, the younger adults scored higher in the relocation memory test than the older adults. More importantly, the instruction manipulation had no effect on the younger adults' performance; however, the older adults scored significantly higher in the intentional than incidental condition. However, because eye movements were not monitored, it is unclear whether or how the instruction manipulation may have affected older adults' object-location memory via modulation of their viewing behaviors. This is particularly relevant since, not only memory, but also eye movements appear to be influenced by factors such as age [21], [22] and task instruction [23], [24].

Clearly, strategies of viewers may determine how they inspect an environment or a scene. Given the positive relationship between viewing behaviors and memory for objects and their locations, it is possible for older adults to improve their object and object-location memory by adopting a suitable viewing strategy. As discussed earlier, remembering is, at least in part, influenced by eye movement behaviors while viewing scenes. It was therefore hypothesized that viewing behaviors and memory performance would vary with instructions, especially for the older participants. To test this possibility, eye tracking technology was applied to an episodic spatial memory paradigm [10], [25].

**Figure 1:**
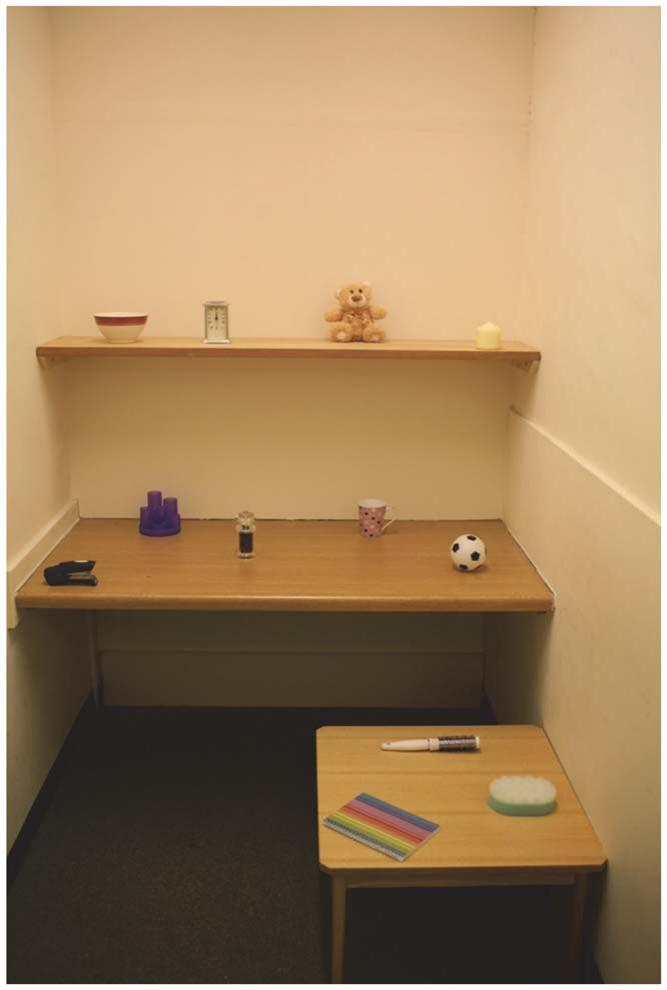
Stimulus photograph used in the present study.

In the present experiment, younger and older adults (45 in each group) viewed a photograph for 10 seconds during which their eye movements were recorded. The photograph depicted a cubicle with 12 objects (targets) pseudo-randomly placed on three different surfaces (Figure 1). Before viewing, some participants were informed of subsequent memory tests (the intentional condition; n = 21 for each age group), while others were not (the incidental condition; n = 24 for each age group). Participants in the incidental condition were told that the study investigated differences in the way that younger and older adults viewed a scene. After viewing, the participants were taken to the actual cubicle depicted in the photograph to complete a recognition task and then a relocation task. They were first presented with 24 objects (12 targets and 12 distractors) from which they selected 12 that they thought were present in the photograph. Next, they rated their confidence about each selection. They were then given the 12 targets to relocate back to where they appeared in the photograph. Afterwards, they rated their confidence about each relocation.

The typical age effect on memory was expected – the younger adults would outperform older adults in memory tests for object identity, spatial location and object location. It was also expected that the older adults (and perhaps the younger adults as well) would perform better in the intentional than incidental condition. More importantly, it was hypothesized that the improved performance in the intentional condition would be related to particular viewing strategies that participants adopted in that condition.

## Results

All measures were submitted to a 2 (age)×2 (instruction) ANOVA unless otherwise noted. All effects were evaluated at a significance level of .05. Significant interaction effects were further assessed by simple main effect tests.

### Recognition task

The results are summarized in Table 1. For both recognition accuracy and confidence ratings for correctly recognized targets, there were significant effects of age group and instruction, but no interaction effect. Recognition was more accurate in the younger than older adults and in the intentional than incidental condition. For correctly recognized targets, one-sample t-tests showed that confidence ratings were significantly above 2 (educated guesses) irrespective of the age group or instruction (of the four groups tested, smallest *t*(23) = 9.86, *p*<.001). However, greater confidence was expressed in the younger than older adults and in the intentional than incidental condition. Nonetheless, confidence ratings for (incorrectly) selected distractors were no different from an educated guess (of the four groups tested, largest *t*(23) = .14, *p* = .89). Thus, although older adults exhibited poorer object memory, they gained equivalent improvement in object memory as the younger adults when the intentional instruction was provided; furthermore, their meta-cognition about the reliability of their object memory appeared as good as that of the younger adults.

**Table 1:** Measures for recognition and relocation tasks and associated ANOVA results.

	Young		Older					
Measure	Incidental	Intentional	Incidental	Intentional	df_Error_	Age group (A)	Instruction (B)	A×B
	M (SE)	M (SE)	M (SE)	M (SE)		F (p)	F (p)	F (p)
P(Recognition)	0.73 (0.02)	0.76 (0.03)	0.63 (0.02)	0.71 (0.03)	86	10.40 (.002)	5.89 (.017)	0.87 (.354)
Confidence: Picked targets	3.37 (0.10)	3.39 (0.07)	2.94 (0.10)	3.32 (0.09)	86	7.46 (.008)	4.74 (.032)	3.84 (.053)
Confidence: Picked distractors	1.98 (0.16)	1.93 (0.15)	1.93 (0.12)	1.87 (0.13)	86	0.14 (.708)	0.13 (.715)	0.00 (.966)
P(Home region)	0.14 (0.02)	0.22 (0.03)	0.08 (0.02)	0.13 (0.02)	86	9.27 (.003)	7.21 (.009)	0.69 (.408)
P(Target region)	0.44 (0.03)	0.44 (0.02)	0.35 (0.02)	0.36 (0.02)	86	12.07 (<.001)	0.02 (.890)	0.02 (.890)
P(Surface)	0.62 (0.02)	0.71 (0.04)	0.53 (0.03)	0.63 (0.04)	86	7.02 (.010)	7.35 (.008)	0.03 (.865)
Displacement-from-home	0.24 (0.01)	0.20 (0.02)	0.28 (0.01)	0.23 (0.02)	86	5.95 (.017)	7.98 (.006)	0.01 (.910)
Best-fit displacement	0.08 (0.01)	0.06 (0.01)	0.14 (0.01)	0.11 (0.01)	86	19.18 (<.001)	4.58 (.035)	0.57 (.451)
Confidence rating when relocating targets at								
Home regions	3.22 (0.17)	3.37 (0.15)	2.83 (0.31)	2.97 (0.31)	64	2.85 (.096)	0.37 (.542)	0.00 (.994)
Other target regions	2.13 (0.17)	2.34 (0.18)	2.18 (0.19)	2.14 (0.17)	83	0.18 (.673)	0.22 (.638)	0.48 (.492)
Unoccupied regions	2.29 (0.15)	2.27 (0.08)	2.06 (0.12)	2.28 (0.12)	86	0.89 (.347)	0.64 (.425)	0.99 (.322)

### Relocation task

Following previous studies [5], [26], several measures were calculated to reflect the degree of (rather than all-or-none) mismatch between actual and memorized states for each relocation [see Methods for their definitions]. Two measures were used to index memory accuracy regarding precise object-location binding: recall probability of home regions P(Home region) and displacement-from-home. Two measures were used to index memory accuracy for the overall spatial layout of the objects within the scene: recall probability of target regions P(Target region) and best-fit displacement. One measure was used to index memory accuracy for binding objects to the three landmarks in the cubicle: recall probability of home surface P(Surface). To be explicit, object identity was taken into account in the computation of P(Home region), displacement-from-home, and P(Surface), but was irrelevant in P(Target region) and best-fit displacement. Greater memory accuracy would be reflected in higher recall probabilities and smaller displacement.

The results are displayed in Table 1. No measure revealed an Age x Instruction interaction (of the five measures, smallest *p*>.4). As expected, the younger adults outperformed the older adults in all relocation measures irrespective of instruction. The results also showed that participants in the intentional condition performed better than those in the incidental condition in all but one measure [i.e., P(Target region)] irrespective of the age group. It appeared that the intentional instruction promoted object-location binding, but did little to enhance the memory for the overall spatial layout of the objects within the scene.

Regarding confidence about relocation accuracy, there was no significant main or interaction effect. The means and SEs of confidence ratings clearly showed that both younger and older adults gave higher confidence ratings when they relocated items back to their home locations than to other target or unoccupied locations. This again shows that older adults' meta-cognition about the reliability of their object-location memory was comparable to that of the younger adults.

### Eye Movement Data

Fixations less than 80 ms or more than 2000 ms were excluded from the analyses. For each target, a region of interest (ROI) was set at 0.75 degrees of visual angle from its edge. Each fixation was allocated to a ROI or to the background (i.e., non-ROI) if it did not land in any ROI. A number of standard eye movement measures were analysed [12], [14]. Gaze (or visit) on a ROI is the sum of consecutive fixations prior to a saccade that leaves the ROI. The measures are listed in Table 2 along with descriptive and *F* statistics.

**Table 2:** Eye movement measures and associated ANOVA results.

	Young		Older					
Measure	Incidental	Intentional	Incidental	Intentional	df_Error_	Age group (A)	Instruction (B)	A×B
	M (SE)	M (SE)	M (SE)	M (SE)		F (p)	F (p)	F (p)
Region of interest (ROI)								
Number of ROIs fixated	10.67 (0.27)	10.10 (0.38)	9.63 (0.36)	10.95 (0.20)	86	0.09 (.768)	1.47 (.229)	9.25 (.003)
Number of ROIs revisited	5.75 (0.40)	5.76 (0.52)	5.54 (0.35)	5.48 (0.45)	86	0.34 (.563)	0.00 (.950)	0.01 (.928)
Number of fixations measures								
Total number of fixations	27.71 (0.99)	27.43 (1.16)	28.67 (1.09)	26.19 (1.14)	86	0.02 (.899)	1.59 (.211)	1.01 (.318)
Total number of fixations on ROIs	23.67 (0.91)	22.48 (1.08)	23.17 (0.89)	22.86 (0.88)	86	0.00 (.950)	0.63 (.428)	0.22 (.641)
Total number of fixations on non-ROI	4.04 (0.64)	4.95 (0.61)	5.50 (0.64)	3.33 (0.55)	86	0.02 (.897)	1.04 (.311)	6.23 (.015)
Total number of visits on ROIs	18.79 (0.74)	18.19 (1.02)	17.96 (0.72)	17.95 (0.71)	86	0.45 (.506)	0.14 (.706)	0.14 (.711)
Mean saccade length (dva)	2.48 (0.13)	2.51 (0.16)	2.14 (0.13)	2.19 (0.15)	86	5.50 (.021)	0.09 (.764)	0.00 (.966)
Fixation duration measures (in ms)								
Total time on ROIs	7765.63 (205.69)	7483.57 (204.50)	7492.63 (189.89)	8265.95 (154.49)	86	1.76 (.188)	1.64 (.204)	7.57 (.007)
Total time on non-ROIs	1129.96 (205.93)	1505.57 (198.88)	1426.21 (156.38)	816.48 (127.99)	86	1.23 (.270)	0.44 (.510)	7.74 (.007)
Mean fixation duration on ROIs	337.17 (14.32)	347.17 (17.58)	335.15 (16.87)	374.89 (18.47)	86	0.59 (.446)	2.19 (.142)	0.78 (.378)
Mean fixation duration on non-ROI	262.97 (19.37)	307.52 (26.24)	265.99 (13.15)	257.00 (23.78)	82	1.31 (.255)	0.74 (.393)	1.67 (.200)
Mean visit duration on ROIs	429.29 (22.15)	436.85 (27.35)	436.36 (25.33)	481.79 (28.68)	86	1.01 (.317)	1.05 (.308)	0.54 (.465)

There was no significant main effect of instruction on any measure. There was a significant main effect of age group on saccade length, indicating shorter saccade lengths in the older than younger adults. Significant Age x Instruction interaction effects were revealed in four measures: the number of ROIs fixated, total number of fixations on non-ROI, total time on ROIs, and total time on non-ROIs. Given the fixed viewing time of 10 seconds, the last two measures were of course highly correlated, *r* = −.91; thus, only the former was further examined. All simple main effects were highly significant (of the 12 tests, smallest *F*(2,43) = 51.3, *p*<.001). That is, the younger adults fixated more ROIs, made fewer fixations on non-ROI, and had longer total viewing time on ROIs in the incidental than intentional condition. However, these trends were reversed for the older adults. In other words, when informed of subsequent memory tests, the older adults spent more time viewing ROIs, resulting in fewer fixations that landed on non-ROI and more ROIs being fixated. That is, the older adults exhibited more object-oriented viewing when they knew that memory about these objects and their locations would be tested.

### Relationship between Viewing and Memory

Analyses thus far were based on participants [i.e., each participant contributed one mean score for each measure]. Following previous studies [16], [21], [27], analyses in this section were based on ROIs [i.e., each participant contributed 12 observations (one for each ROI) for each measure] and the relationships were examined via regression analyses for each age group. Measures commonly used to index object-oriented viewing are the number of fixations on an ROI, the number of visits on an ROI, and the total fixation time on an ROI [16], [21], [27]. In this set of analyses, all three measures gave identical pattern of results, which is not surprising because they were highly correlated (*r* = .80∼.91, *p*<.001). For simplicity, the three measures were aggregated into one composite viewing score using the Horst method [28]. Three dependent measures were respectively examined – whether the target contained in a ROI was correctly identified or not (a binary variable) indexing object memory, displacement-from-home indexing object-location memory, and best-fit indexing location memory.

Although the main interest was the viewing-memory relationship, object-oriented viewing might be affected by instructions as shown in the previous section. Furthermore, some participants might influence the relationship more than other participants. To remove these effects, the instruction factor was entered into the model first and followed by a set of dummy variables (number of participants minus one) that categorically coded the participants. As in Williams et al. [27], *F* values for changes in *R^2^*attributable to the viewing score are reported.

There were significant point-biserial correlations between viewing and object memory accuracy (a binary variable – target recognized or not; thus, point-biserial correlations were used) for both younger and older group, *r*_pb_ = .158 and .223, *F*_change_(1,494) = 7.88 and 16.34, *p = *.005 and *p*<.001. The strength of association did not differ between the two age groups (z = 1.11, *p* = .27). There were also significant Pearson correlations between viewing and accuracy in object-location memory (measured by displacement-from-home) for both younger and older group, *r* = −.197 and −.217, *F*_change_(1,494) = 12.95 and 16.30, *p*s<.001. Again, there was no group effect on the strength of association (z = 0.34, *p* = .73). However, there was no significant correlation between viewing and accuracy in location memory (measured by best-fit displacement) for either group, *r* = −.057 and −.005, *F*_change_(1,494) = 1.06 and 0.009, *p*s>.3. Therefore, objects and their associations to locations were remembered better if they were viewed more frequently or longer; however, such object-oriented viewing did not appear to promote memory about general layout of the environment.

### Participant characteristics

Table 3 summarizes characteristics of the sample as a function of age group and instruction. An interaction effect was revealed in the threshold for discriminating spatial pattern at high spatial frequency (i.e., 18 cycles per degree), showing participants had lower threshold in the intentional than incidental condition for the younger group; however, this pattern reversed for the older group. Main effects of age group emerged in all but two measures – the younger group had more years in education, scored higher on the short-term visual recall span, but had lower IQ measures. As expected, the younger adults had better visual-sensory abilities – they suffered less acuity loss and had lower thresholds for discriminating spatial patterns across low to high spatial frequencies. An instruction effect was revealed in two measures, indicating the participants in the incidental groups had slightly more years in education and scored slightly higher in the NART than those in the intentional condition. Thus, the present results showing better performance in the intentional condition were unlikely due to confound of cognitive abilities.

**Table 3:** Participant characteristics as a function of age group (A) and instruction condition (B) and the results of A×B ANOVA.

	Young		Older					
Measure	Incidental	Intentional	Incidental	Intentional	df_Error_ d	Age group (A)	Instruction (B)	A×B
	M (SE)	M (SE)	M (SE)	M (SE)		F (p)	F (p)	F (p)
Sample size [No. males]	24 [4]	21 [8]	24 [2]	21 [7]				
Age (years)	22.08 (0.65)	20.10 (0.31)	71.17 (1.25)	71.67 (1.38)	86	2520.75 (<.001)	0.55 (.460)	1.54 (.218)
Education (years completed)	15.67 (0.56)	14.52 (0.32)	14.38 (0.57)	13.40 (0.59)	86	5.19 (.025)	3.99 (.049)	0.03 (.871)
Cognitive measures								
Short-term visual recall span	10.17 (0.39)	9.67 (0.43)	8.10 (0.34)	7.86 (0.41)	86	24.57 (<.001)	0.89 (.348)	0.11 (.739)
Forward digit span	6.88 (0.20)	6.62 (0.23)	6.75 (0.21)	6.76 (0.25)	86	0.00 (.968)	0.30 (.586)	0.36 (.550)
Backward digit span	4.75 (0.22)	5.29 (0.28)	5.17 (0.29)	5.29 (0.27)	86	0.61 (.436)	1.51 (.222)	0.61 (.436)
National Adult Reading Test	102.94 (1.65)	98.40 (1.53)	114.78 (1.12)	112.14 (2.14)	73^e^	57.70 (<.001)	4.55 (.036)	0.32 (.573)
Spatial vision								
Acuity loss (LogMAR)^a^								
Near vision	0.11 (0.03)	0.12 (0.02)	0.33 (0.04)	0.28 (0.02)	86	49.53 (<.001)	0.35 (.553)	1.34 (.250)
Distant vision	0.14 (0.03)	0.05 (0.02)	0.18 (0.04)	0.16 (0.03)	86	6.21 (.015)	3.02 (.086)	1.42 (.237)
Contrast threshold (dB)^b^								
2 cycles per degree (cpd)	−9.04 (0.85)	−8.67 (0.69)	−3.23 (1.06)	−3.59 (1.23)	79	29.92 (<.001)	0.00 (.994)	0.13 (.716)
6 cpd	−2.97 (1.18)	−3.91 (1.43)	3.22 (1.72)	4.47 (1.06)	79	27.72 (<.001)	0.01 (.914)	0.63 (.431)
12 cpd	9.67 (1.55)	4.90 (1.07)	17.25 (2.25)	18.30 (1.32)	79	40.27 (<.001)	1.27 (.262)	3.10 (.082)
18 cpd	21.30 (1.60)	15.77 (1.31)	25.55 (2.01)	30.82 (2.08)	79	28.45 (<.001)	0.00 (.945)	8.91 (.004)
Composite score of visual ability^c^	−8.00 (2.53)	−13.73 (1.86)	9.04 (3.71)	10.95 (2.29)	79	57.19 (<.001)	0.48 (.491)	1.92 (.170)

a The log of the minimum angle of resolution; 0 means no loss, positive values indicate vision loss, and negative values indicate normal or better visual acuity.

b Thresholds were measured in dB; the greater the threshold, the poorer the sensitivity.

c A low score indicates better ability than a high score.

d The variation in the degrees of freedom in the error term reflects missing data.

e Only included those whose first language is English.

## Discussion

In the current study, eye movements of younger and older participants were recorded while they viewed a photograph of a room with 12 objects located on three surfaces. They viewed it with or without being instructed that memory for these objects and their locations would be tested (intentional vs. incidental). The data were analyzed to consider visual encoding processes in relation to memory for objects and their locations. There were three main findings: First, memory changed with instruction and age. Second, viewing behavior changed with instruction and age. Third, there is a fundamental relationship between what is viewed and what is remembered, in terms of memory for object identity and object location but not overall positional configuration.

As expected, the older adults performed worse in both recognition and relocation tasks than the younger adults, indicating poorer object (identity) memory, location and object-location memory in the older adults. Both younger and older adults performed better in the intentional than incidental condition. Furthermore, increased viewing on ROIs was associated with more accurate object and object-location memory for both age groups and the strength of association appeared independent of the age group. These findings are consistent with previous work [16], [21], [27] and demonstrate that direct fixation of objects is important for accurate memory of their identities and locations. Furthermore, the more time spent viewing an object, the better the memory record. However, the results showed that increased viewing was not associated with improved memory for overall spatial location. Again this is consistent with previous work, in which it is postulated that location memory is relatively automatic, with a spatial map of a scene being created during the first few fixations on a scene [16], [29], [30]. Accordingly, the type of viewing behavior engaged in after these initial fixations is inconsequential to location memory as the map has already been formed.

More importantly, the present study enabled examination of whether environmental support in the form of explicit instructions may help older adults to overcome memory deficits by using strategies and/or constructive operations as per Craik [1]. Indeed, the older adults exhibited more memory-enhancing viewing behaviors in the intentional than incidental condition – they spent more time viewing ROIs, resulting in fewer fixations landing on non-ROI and resulting in more ROIs being fixated. By contrast, the younger adults showed the opposite trend in the viewing behaviours, fixating fewer ROIs and made more fixations on non-ROIs and had longer total viewing times on non-ROIs in the intentional than the incidental condition. Somewhat surprisingly, however, they still performed better in the intentional than the incidental condition. Nonetheless, the finding was not implausible. Although the younger adults showed an increase in the fixations on non-ROIs in the intentional condition, this does not necessarily mean that they spent more time exploring non-ROIs. It is well established that younger adults have wider functional field of view (i.e., the spatial area needed to successfully perform a visual task without moving eyes or head), which may extend up to 5 degrees of visual angle [31]. The largest center-to-center distance between two adjacent objects in the photograph was within 5 degrees and the distance between adjacent surfaces was within 4 degrees. It is possible that younger adults sometimes fixated at a location between two objects to allow them to inspect both at the same time. This possibility remains to be investigated.

One conclusion that can be drawn from these findings is that older adults may improve their memory for objects, locations and object locations when they use appropriate strategies and/or constructive operations [1]. Thus, the findings are consistent with the claims of Craik [1] in that appropriate environmental support facilitates memory in older adults. A second important point of note from these findings is that although memory performance of older adults improved, it still did not reach the level observed in younger adults. Thus, whilst environmental support can facilitate memory, it does not completely eliminate deficits due to age. Another point that may be relevant to this finding is the fact that the viewing duration in the present study was fixed at 10 seconds, and this relatively short period could have limited the scope for improvement in the older adults. A wide range of experimental paradigms has demonstrated that the speed of information processing is slower in older than younger adults [32] and the degree of slowing appears greater in the spatial than linguistic domain [33], [34]. Had a longer viewing time been used, memory performance of the older adults may have attained the level observed in the younger adults. This possibility remains to be investigated. Nonetheless, the fact remains that the present results clearly indicate that older adults' object and object-location memory can be improved via appropriate viewing behaviours.

In conclusion, the present study investigated whether variability in encoding behavior (caused by instruction) could account for variability in recall behavior in older and younger adults. Importantly, the results suggest that there is a fundamental relationship between eye movements and memory for object identity and object locations, and that variability in encoding behavior accounts for, at least some, variability in memory performance. Specifically, instruction at encoding affects viewing behavior differentially for younger and older adults, and this leads to increases in both object identity and object location memory for both age groups. Furthermore, variability in viewing behavior did not impact on memory for the overall spatial layout of the scene, suggesting that encoding behaviors that underpin object identity and object-location memory are different to those underpinning memory for global spatial layout. It is encouraging to observe that older adults can improve their object and object-location memory if they spend more time encoding relevant visual information. Further research is required to determine whether or not it is possible for healthy older adults to achieve the same level of memory performance as younger adults by further lengthening the visual encoding period, and more specifically, the time spent directly fixating objects in a scene that are to be remembered.

## Methods

The study was approved by the Ethics Committee of Psychology, University of Southampton. All participants were treated according to the ethical standards of the British Psychological Society. All participants gave written informed consent.

### Participants

The data of 45 younger adults and 45 older adults were analyzed. They received standard written and verbal instructions. The younger adults were students of the University of Southampton and took part voluntarily or to partially fulfill course requirements. The older adults were recruited through the Older Adult Volunteer List, School of Psychology, University of Southampton. Participants were tested individually in one session lasting between 40 minutes to 1 hour.

### Apparatus and Materials

Stimuli included a cubicle (Width×Depth: 1.7×2.76 m) and 24 objects (12 targets and 12 distractors). The cubicle contained three surfaces (top shelf, middle desktop, and bottom coffee table). The targets (a bowl, alarm clock, teddy bear, candle, stapler, desk tidy (pen holder), pepper grinder, mug, small ball, hair brush, sponge, notebook) were comparable in size to the distractors (a drink bottle, teapot, small box, torch, camera, rubix cube, martini glass, ball of string, pair of sunglasses, remote control, trowel, corkscrew).

The 12 targets were pseudo-randomly located over the three surfaces in the cubicle and a digital photograph was taken (Figure 1). The photograph was presented on a 24-inch monitor (resolution: 1024×768 pixels). The photograph measured 18.8 cm (W) and 29 cm (H) cm on the monitor and subtended 16°×25° of visual angle at a viewing distance of 66 cm, maintained by a chin and forehead rest. Visual angles of the targets (width or height) varied from 0.43° to 2.52° (*M* = 1.14°, *SD* = 0.48°). ROIs were the 12 target objects and were set at ∼.75 degrees of visual angle from the edges of each object. Eye movement data were recorded using the EyeLink 2000 eye tracking system (SR-Research Ltd., Toronto).

Snellen charts were used to measure visual acuity at near (16 inches) and far (3 feet) distances. A computerized orientation identification task (via two-alternative forced-choice) was used to estimate contrast threshold at spatial frequency 2, 6, 12 and 18 cycles per degree. To appraise short-term memory and concentration, WAIS-III© forward and backward digit span [35] and the Visual Patterns Test [36] were administered. The National Adult Reading Test (NART) was used to estimate premorbid intelligence level [37], and, for older adults only, the Montreal Cognitive Assessment (MoCA) [38] was used to detect mild cognitive impairment.

### Design and Procedure

There were two independent variables: age (younger vs. older adults) and instruction (incidental vs. intentional). After calibrations for eye movements, participants viewed the digital photograph for 10 seconds with both eyes although only movements of the right eye were recorded. In the intentional condition, participants were explicitly told to view the scene to be ready for subsequent memory tests, whereas in the incidental group, no warning was given about the memory tests. Participants in the incidental condition were told that the study investigated differences in the way that younger and older adults viewed a scene. After viewing, participants in the incidental condition were then told the true purpose of the study and were asked to give consent to take part in the memory tests. All participants were then taken to the actual cubicle shown in the photograph for recognition and relocation tasks.

For the recognition task, targets and distractor objects were pseudo-randomly displayed on the desk and participants were required to select the 12 targets from the distractors. After 12 objects had been selected, participants gave confidence ratings for each object selected (in order of selection) on a scale of 1 to 4, where 1 = complete guess, 2 = educated guess, 3 = fairly confident, and 4 = very confident. They were then given the 12 targets (and the distractor items were removed) and were asked to relocate each object back to the position that it was located in the photograph. After relocating all targets, they again used the 4-point scale to rate their confidence of each replacement (rated in order of replacement). Participants then completed a battery of tasks designed to assess general visual-sensory and cognitive abilities.

### Dependent Measures for the Relocation Task

Scoring for the relocation task was not as straightforward as that for the recognition task because “location memory is often imprecise, and much recall is seen as near-miss errors” ([39], p. 67). Furthermore, it is important to distinguish the two types of spatial location memory: memory for the location of individual objects in a scene (or object-location memory) and memory for occupied locations in the scene, regardless of correct object identity (or location memory) [5]. Thus, scoring allowed for some degree of imprecision and measures were to reflect the degree of (rather than all-or-none) mismatch between actual and memorized states for each recall, similar to those used by Postma and colleagues [26].

The room was divided into 33 regions – 1×6 regions on the top shelf, 3×6 regions on the middle desktop, and 3×3 regions on the bottom coffee table. A region measured about 23 cm×30 cm (Width×Depth) on the top surface, 23 cm×23 cm on the middle surface, and 30 cm×30 cm on the bottom surface. Each target occupied a unique region – a target's *home* region. Hence, there were 12 occupied (or home) regions and 21 unoccupied regions. A target's relocation was categorized into a region as a target's *located* region. Thus, a target's located region could be its home region, a region of another target, or an unoccupied region.

To respectively index the accuracy of memory for an object in a precise location (object-location binding), for an object to its correct surface (a form of topology binding), and memory for the overall spatial layout of the objects within the scene, the following measures were computed: recall probability of home regions or P(Home region), of objects' original surfaces or P(Surface), and of target (including home) regions regardless of object identity or P(Target region) for each participant.

Next, a displacement-from-home measure was computed to evaluate the imprecision of object-location memory. Similar to the absolute error defined in Postma et al. [26], coordinate displacement for each target with respect to the coordinate system of the 2D stimulus photograph was calculated. The coordinate displacement of a target is the Euclidean distance between centres of the target's home and located regions divided by the maximum possible value; thus, 0 means perfect relocation, while 1 means maximum displacement. However, the task in Postma et al. involved a 2D space, while the present study involved a 3D room; moreover, in the present study the between-surface distances were shorter than many between-region distances on the same surface. To ensure an error measure was smaller for targets relocated to a correct than incorrect surface, displacement-from-home was defined by the average of coordinate displacement and categorical displacement (see below). To calculate categorical displacement of a target, an ordinal value was assigned to each of the three surfaces (1, 2, and 3), and the absolute difference between the target's original and located surface was divided by 2 (the maximum possible difference) to normalize the displacement value between 0 and 1. Thus, the value of displacement-from-home varied between 0 and 1.

Following Postma et al. [26], best-fit displacement was also calculated to assess memory imprecision in the overall positional configuration. For each participant, one-to-one associations were made between located and target regions so that the mean displacement (which was the average of categorical and coordinate displacements) across the 12 targets was minimized. Thus, best-fit displacement gives a measure of positional memory – it ignores object identity and reflects the degree to which targets have been placed close to occupied regions.
